# Relay Intercropping Winter Cover Crop Effects on Spring Forage Potential of Sweet Maize Stover and Yearling Cattle Beef Performance

**DOI:** 10.3390/ani12151923

**Published:** 2022-07-28

**Authors:** Leonard M. Lauriault, Steven J. Guldan, Fernanda G. Popiel-Powers, Robert L. Steiner, Charles A. Martin, Constance L. Falk, Mark K. Petersen, Tammy May

**Affiliations:** 1 Rex E. Kirksey Agriculture Science Center, New Mexico State University, Tucumcari, NM 88401, USA; 2 Sustainable Agriculture Science Center, New Mexico State University, Alcalde, NM 87511, USA; sguldan@nmsu.edu (S.J.G.); fpowers22@gmail.com (F.G.P.-P.); cmartin@nmsu.edu (C.A.M.); 3 Department of Economics and International Business, New Mexico State University, Las Cruces, NM 88003, USA; rsteiner@nmsu.edu; 4 Department Agricultural Economics and Agricultural Business (Emeritus), New Mexico State University, Las Cruces, NM 88003, USA; cfalk@nmsu.edu; 5 USDA-ARS Fort Keogh Livestock and Range Research Laboratory (Retired), United States Department of Agriculture (USDA), Miles City, MT 59301, USA; bearpause2@gmail.com; 6 Animal and Range Sciences Department, New Mexico State University, Las Cruces, NM 88003, USA; tamalita.may@gmail.com

**Keywords:** forage, sweet maize, cereal rye, hairy vetch, relay intercropping, grazing, nutritive value, crude protein, in vitro dry matter disappearance

## Abstract

**Simple Summary:**

Maize stover is used globally as winter feed for livestock but the nutritive value is low, requiring supplementation. Small landholders who grow sweet maize for the fresh produce market often also have cattle with little access to winter forage. Grazing cover crops with the stover can potentially increase the available nutritive value. Intercropping the cereal rye or hairy vetch into sweet maize approximately 65 days after planting (V7–9 sweet maize maturity) did not influence sweet maize stover biomass yield or nutritive value after winter. However, the crude protein concentration of hairy vetch was greater than rye, but digestibility was not different. Average daily gains by yearling cattle were similar when grazing maize–rye or maize–vetch. Producers with limited land resources should consider the timing of the spring planting of the primary crop, in this case, sweet maize, in relation to the initiation of grazing of winter cover crops to maximize utilization of the previous crop’s residue (stover) as well as the cover crop itself before the primary crop planting time.

**Abstract:**

Small landholders who grow sweet maize for the fresh produce market often also have cattle with little access to winter forage. Grazing cover crops with sweet maize stover can potentially increase the available nutritive value. A 3-year randomized complete block study with three replicates at New Mexico State University’s Alcalde Sustainable Agriculture Science Center compared sweet maize (*Zea mays* var. *rugosa*) with sweet maize relay intercropped at the V7–9 stage with cereal rye (rye: *Secale cereale* L.) or hairy vetch (vetch: *Vicia villosa* Roth) for early spring grazing. Intercropping the rye or hairy vetch into sweet maize did not influence the sweet maize stover biomass yield or nutritive value after the winter. The dry matter (DM) yield and crude protein (CP) concentration of hairy vetch biomass was greater (*p* < 0.01) than rye biomass (1.46 vs. 2.94 Mg DM ha^−1^ for rye and hairy vetch, respectively, and 145 vs. 193 g CP kg^−1^ for rye and hairy vetch, respectively). Average daily gains by yearling cattle were not different when grazing maize–rye or maize–vetch. Producers should consider the spring planting timing of the primary crop and the initiation of grazing in the winter or the spring to maximize the utilization of the previous crop’s residue (stover), as well as the cover crop itself.

## 1. Introduction

Improved productivity of available land resources is a global concern [1]. Moreover, farmers seek options to sustain their livelihood in the face of increased input costs, while protecting soils and other natural resources [2]. Crop residues, such as maize stover, help to protect the soil, but also have been used as an economical winter feed source for livestock in many parts of the world, either grazed in situ [1,2,3,4], or as stored feed ex situ [2,5,6]. Grazing is probably the best use of maize stover, although energy and/or protein may be limited, except possibly for non-lactating cows or calves, especially in irrigated fields [4,7]. Klopfenstein et al. (1987) [7] reported whole plant maize CP and in vitro dry matter digestibility (IVDMD) of 48 and 566 g kg^−1^, respectively.

Cover cropping is another management option to conserve the water and soil and to reduce the cost of applied inorganic fertilizers [8,9,10]. Legumes are used to acquire and store atmospheric nitrogen and non-leguminous cover crops can be used to recover and store applied nutrients [11]. Grazing cover crops has the potential of providing both cash returns and soil quality improvements [8,10,11]. Consequently, interest is increasing in grazing cover.

Intercropping and sequential cropping describe multiple cropping options commonly used in warmer climates to increase the productivity per unit of land [12,13,14]. Relay intercropping is a form of multiple cropping in which a sequential crop is planted when the first crop has reached its reproductive stage [12]. This reduces the competition between the primary (generally the higher value crop and planted first without competition) and secondary crop, as opposed to planting the secondary crop earlier [1]. Relay intercropping is suitable in some of the cropping scenarios, but not others. When the primary crops were not stressed, relay intercropping cool-season annual and perennial legumes into standing sweet maize to increase the available autumn forage generally led to no reduction in the primary crop yield [9,15].

Winter cereal forages are used widely in the irrigated western USA and other semiarid regions of the world for supplemental autumn and winter forage, and are especially beneficial to provide pasture high in nutritive value for recently weaned calves [3,16]. Lauriault et al. (2018) [17] reported that newly weaned beef cattle grazing sweet maize stover overseeded with oat (*Avena sativa* L.) or turnip (*Brassica rapa* L.) in the autumn had increased average daily gains (ADG), compared to those grazing sweet maize stover only. Butler et al. [18] reported a similar performance by cattle grazing rye-annual ryegrass (*Lolium multiflorum* Lam.) pastures, when either nitrogen fertilized or grown with a mixture of multiple winter annual legumes, including hairy vetch. They [18] reported cereal rye CP and total digestible nutrients (TDN) of 151 and 822 g kg^−1^, respectively, and CP and TDN of 261 and 840 g kg^−1^, respectively, for the hairy vetch.

A sweet maize–legume relay intercropping system previously described [15] was not evaluated for its forage nutritive value, and others have stated that intercropping forages into annual cash crops requires more information [1]. Therefore, the objectives of this research were to determine how relay intercropping rye or hairy vetch might have an effect on the sweet maize stover component and the overseeded forage (rye or hairy vetch) biomass and nutritive value and cattle performance.

## 2. Materials and Methods

### 2.1. Experimental Site Description

The site and general methods for this study were previously described in Lauriault et al. [17], using different experimental units within the same randomization. In the present study, the trials with relay intercropping treatments (sweet maize overseeded with rye (maize–rye) and sweet maize overseeded with hairy vetch (maize–vetch)) were conducted in three years at New Mexico State University’s Alcalde Sustainable Agriculture Science Center (36.08° N, 106.05° W, elev. 1745 masl). The weather data were collected from a station located within 1 km of the study site. In Table 1, Years 1 to 3 refer to the years in which the grazing was conducted and the preceding year is when the sweet maize production took place and the intercrops were sown overseeded. The autumn grazing study reported here [17], using different experimental units than the spring grazing study, was conducted in Years 1 and 3 of the present study, but in the previous report, they were labeled as Years 1 and 2.

### 2.2. Crop Establishment and Management in Summer and Autumn

The same field was used for Years 1 and 3 of the study, with Year 2 conducted in an adjacent field because the grazing extended beyond sweet maize planting time each year. The sweet maize management and overseeded crop planting technique were previously described [17]. For the present study, the rye and hairy vetch were broadcast seeded (128 and 45 kg ha^−1^ seeding rates, respectively) in late June/early July with the last cultivation of the sweet maize. The remnant stover was left intact over the winter, with one irrigation applied before the commencement of grazing each year to promote the growth of the overseeded crops. No additional fertilizers were applied specifically for the overseeded crops.

### 2.3. Forage Sampling in the following Spring

The forage samples were collected (14, 20, and 15 April in Years 1 to 3, respectively) from three 3 m × 1.8 m locations within each plot. At the sampling time, the maize stover was weathered while the rye had headed and the hairy vetch was still pre-bud. Whole maize plants were cut with a machete 5–10 cm above the soil surface, counted and weighed fresh in the field, after which the ears that were not previously market ready and the leaves were stripped and weighed separately. The stalk yield was determined by the difference. While the leaf sheaths may or may not have been stripped with the leaves, it was assumed that stripping the leaves in this way would simulate removal by grazing livestock [19], as opposed to harvesting only the leaf blades. The whole plant sweet maize biomass DM was calculated as the sum of the component DM biomasses.

The overseeded rye and hairy vetch biomass was then harvested to ground level with hand-held shears and weighed fresh in the field. The forage samples were also taken after the grazing was terminated, near the locations previously sampled to determine post-grazing DM yield and the disappearance was calculated as the proportion of the forage that had disappeared during the grazing occupation [4]. A subsample of each sweet maize stover component (ears, leaves, and stalks) and overseeded forage component collected from each of the harvested areas before and after grazing was dried in a forced-air oven at 65 °C for 48 h for moisture determination and biomass DM yield calculations. Pre-grazing subsamples were ground to pass a 1-mm screen. The laboratory analyses were conducted to determine the crude protein (CP) concentration by Kjeldahl [20] and 48-h in vitro DM disappearance (48-h IVDMD) [21]. Sweet maize whole plant CP and 48-h IVDMD concentrations were calculated as the weighted mean of the ear, leaf, and stalk components.

### 2.4. Grazing Management and Cattle Measurements in Spring

The grazing management and cattle measurements were previously described [17]. For the spring forage treatments, yearling beef heifers (predominantly British x continental cross) (177 ± 8 kg BW (body weight), 172 ± 9, and 202 ± 10 kg BW in Years 1 to 3, respectively) grazed the intercropping treatment-paddocks. Prior to grazing, all of the animals were acclimated using alfalfa hay. Grazing was initiated on 14, 20, and 15 April of Years 1 to 3, respectively, and the animals were removed on 16, 26, and 27 May of Years 1 to 3, respectively, when the overseeded rye forage had been effectively utilized. Prior to the onset of grazing and when grazing was terminated, the heifers were shrunk overnight and weighed to calculate the average daily gain (ADG).

### 2.5. Statistical Analyses

The statistical analysis was as previously described [17], such that each test was a randomized complete block with three replications, and the subsample and cattle data within each plot were averaged to represent that plot. The pre-grazing biomass and pre-grazing CP concentration and 48-h IVDMD and post-grazing biomass and the disappearance of the sweet maize stover ear, leaf, and stalk component data were analyzed with the mixed procedure of SAS (v. 9.4, SAS Inst., Inc., Cary, NC, USA, 2013); however, the 48-h IVDMD data for Year 1 were lost shortly after the laboratory analysis. Consequently, only the data for Years 2 and 3 are included in the analysis and the results for that variable. The tested effects included year, sweet maize stover component (ear, leaf, and stalk), relay intercropping treatment (maize–vetch and maize–rye), and all of the possible interactions. Pre-grazing whole plant nutritive value, pre- and post-grazing whole plant biomass and the disappearance of sweet maize stover; the pre-grazing nutritive value and pre- and post-grazing biomass and the disappearance of overseeded forage components; initial cattle weights; and cattle ADG were analyzed with the Mixed procedure of SAS (v. 9.4, SAS Inst., Inc., Cary, NC, USA, 2013). The tested effects included relay intercropping treatment and year and their interaction. Rep × year and residual mean squares were considered random and used as the denominators for tests of significance. When a main effect or interaction was significant, protected (*p* ≤ 0.05) least significant differences were used to determine where the differences occurred among the treatment means. All of the differences reported are significant at *p* ≤ 0.05.

## 3. Results and Discussion

### 3.1. Sweet Maize Stover Biomass

Sweet maize stover components were all different from each other in biomass yield when measured (0.25, 0.44, and 1.23 Mg DM ha^−1^ for the ear, leaf, and stalk components, respectively; standard error of the difference between lsmeans (SED) = 0.04, *p* < 0.0001), but there were no interactions with the year and/or the overseeded crop. Although the rankings were consistent with the sweet maize stover biomass yield measured in the autumn [17], these values were about 60% of the ear and leaf and 75% of the stalk biomass for the maize alone in the autumn grazing study, probably due to weathering over the winter [4]. There was no year or relay intercropping treatment effect or interaction for the sweet maize stover total DM biomass (Table 2).

### 3.2. Overseeded Forage Biomass

A year × intercropping treatment interaction of magnitude existed for the overseeded forage biomass that was an interaction of magnitude that was different across the years, but consistent within each year to the intercropping treatment main effect differences (Table 2), except that there was no difference between the intercrops in Year 3, due to a considerably lower yield of hairy vetch that was not different from rye. In the present study, the Year 3 overseeded species’ growing season was warmer and had greater precipitation than previous years (Table 1). Cicek et al. [10] reported reduced hairy vetch yields due to excessive soil moisture in two out of three years of their study. That also could have been a factor in the present study, when greater precipitation fell in the autumn and spring growth periods of Year 3 (Table 1), coupled with the clayey soils, such as those in the present study. Guldan et al. [15] reported hairy vetch yields of 1.78 and 3.81 Mg ha^−1^ in a 2-year study at this same location, although they did not state any suspected cause of the difference and the historical weather data revealed little difference in the temperature or precipitation and the plots were furrow-irrigated as needed throughout the growing season. Cicek et al. [10] and others [as cited by 15] have also reported erratic performance by cool-season annual legumes due to environmental influences, especially compared to grasses [9]. Lauriault and Kirksey [22], at a more southerly location and lower elevation, reported monoculture cereal rye yields about twice those of the present study (Table 2) when planted a month later and harvested a week earlier at the same stage of maturity. In that study [22], the rye yields were not reduced by intercropping with hairy vetch or winter pea (*Pisum sativum* subsp. *arvense* (L.) Poir).

### 3.3. Sweet Maize Stover CP Concentration

Whole plant sweet maize stover CP concentration was similar across the years and relay intercropping treatments and there was no interaction between those effects (Table 2); however, there were differences in the sweet maize component CP and for all of the interactions, except the intercropping treatment x sweet maize component. The year × intercropping treatment × sweet maize component interaction was largely driven by the greater ear CP for the sweet maize overseeded with rye compared to the ear CP of maize interseeded with hairy vetch in Year 1, when there were no other differences within any sweet maize component in any year (data not shown). Consequently, the mean CP concentration among the sweet maize stover components was 107, 87, and 119 g kg^−1^ for the ear, leaf, and stalk components, respectively (SED = 3, *p* < 0.0001) with all being different from each other. These values are approximately 88% of those reported for sweet maize ear and stalk in the autumn [17], while there was little change for leaf CP from the autumn through the spring (89 g kg^−1^ in the autumn [17]). Little information about the sweet maize stover nutritive value was available in the literature, and none for the post-winter stover.

### 3.4. Overseeded Forage CP Concentration

There were no significant year or year × relay intercropping treatment effects for the overseeded forage biomass CP concentration; however, there was a difference for the main effect of the relay intercropping treatment because the hairy vetch biomass had a greater CP concentration than the rye biomass (Table 2). This, again, may have been related to species differences, as rye is a grass and hairy vetch is a legume with symbiotic nitrogen-fixing capability [10].

### 3.5. Sweet Maize Stover 48-h IVDMD

Recall that only Years 2 and 3 data were available for 48-h IVDMD. A year × sweet maize component interaction of magnitude existed for 48-h IVDMD across the years because in Year 2, with the stalk 48-h IVDMD intermediate to and not different from either the ear or leaf, the ear and leaf differed from each other; but, in Year 3, all of the components were different from each other (data not shown). Regarding the component main effect differences (Table 2), similar to the difference among the sweet maize components in CP concentration, the 48-h IVDMD of sweet maize stalks was greater than the ears and leaves, which also were different from each other (540, 509, and 571 g kg^−1^ for the ear, leaf, and stalk components, respectively; SEM = 9, *p* < 0.0001). The difference was consistent with the report by others [4]. While the rankings were consistent with the measurements in the autumn [17], leaf IVDMD was ~45% more digestible than before the winter, while the ear and stalk components were only 11 and 2% different, respectively. As mentioned, the low winter precipitation in continental semiarid environments in most years (Table 1) should minimize the leaching of water soluble constituents that would increase the fiber proportion and reduce digestibility rather than increase it.

There was no difference between the relay intercropping treatments for the whole plant sweet maize stover 48-h IVDMD (Table 2). In the autumn forage study previously reported [17], the turnip improved the IVDMD of sweet maize stover compared to the maize alone or the maize intercropped with oat. That effect was attributed to the additional hydraulic lift [13,23,24] and rhizosphere pH modification by *Brassica* spp. [14], that increases the availability of soil P and access to soil water to increase the uptake by the sweet maize [25,26], which reduces fiber components [24]. In turn, fiber has an effect on digestibility [27]. No reports were found in the literature regarding the hydraulic lift by hairy vetch as there was for Brassicas, oat, and rye [23].

### 3.6. Overseeded Forage 48-h IVDMD

Relay intercropping had no effect on the overseeded forage biomass 48-h IVDMD but the year and year × intercropping treatment interaction effects were significant (Table 2). The interaction was due to a difference in magnitude among the years, such that rye had greater digestibility in Year 3 than in Year 2, while the hairy vetch digestibility did not change across the years. This was not unexpected because the cereal forage nutritive value declines with maturity, especially after heading [28]. In the studies slightly south of the present study location, rye initiated heading on about 7 April [29] and hairy vetch did not initiate flowering until 26 April [30]. In the present study, the rye in Year 2 was sampled about two weeks after heading (20 April), but only 1 week after heading (15 April) in Year 3, and sampling took place each year before the hairy vetch initiated flowering.

### 3.7. Post-Grazing Sweet Maize Stover Biomass and % Disappearance

While post-grazing sweet maize biomass and disappearance did not differ among the relay intercropping treatments (Table 3), the post-grazing biomass did differ among the stover components because the stalk post-grazing biomass was greater than that of ear and leaf, which were different from each other (0.10, 0.21, and 0.76 Mg DM ha^−1^ for the ear, leaf, and stalk components, respectively; SEM = 0.05, *p* < 0.0001). The year × maize component interaction was significant for the post-grazing biomass because stalk biomass was different each year, being not different from leaf biomass in Year 1 to increasing each year thereafter (data not shown). The ear and leaf post-grazing biomass remained not significantly different within or across years (data not shown), but the increasing stalk biomass each year contributed to a significant difference in the post-grazing whole plant stover biomass and disappearance from Year 1 to Year 2 (Table 3). The percentage disappearance also differed among the sweet maize stover components because the disappearance of the stalk was less than the disappearance of the other two components (60, 51, and 39% disappearance for the ear, leaf, and stalk components, respectively; SEM = 6, *p* < 0.0031).

The sweet maize stover disappearance in the present study was considerably less than when the sweet maize stover was grazed in the autumn [17]. There was approximately 28% less initial maize biomass than in the spring-grazed plots (1.90 Mg ha^−1^ in Table 2 vs. 2.64 Mg ha^−1^ for intercropped initial sweet maize biomass in the autumn [17]). There was an approximately 12-d longer grazing period in the autumn to effectively utilize the available oat and turnip forage to a level at which availability would become limited [31], compared to the 33-d average grazing occupation in the present study to utilize the rye to the same level (0.4 Mg ha^−1^, Table 2 [17]), which likely contributed to the difference in the sweet maize stover disappearance between the spring grazing reported here and the autumn grazing previously reported [17].

### 3.8. Post-Grazing Overseeded Forage Biomass and % Disappearance

The year and relay intercropping treatment effects were not significant for the post-grazing overseeded forage biomass and there was no interaction (Table 3). The rye was grazed to a point of limited availability [31], similar to the oat and turnip that were grazed in the autumn [17], while the hairy vetch was not grazed to that extent in the spring, although, almost twice as much of the initial hairy vetch forage disappeared than the rye forage (Table 2 and Table 3). However, because the initial hairy vetch biomass was greater, the disappearance percentage was less. Consequently, with a greater biomass after grazing in this study for both sweet maize and hairy vetch, the maize–vetch could have been grazed longer than the maize–rye. Alternatively, allowing the hairy vetch to progress through seed production as a living mulch would further protect the soil from erosion due to tillage and could lead to stand naturalization as a nitrogen-providing grazable cover crop [9,10,11,30].

Butler et al. [18] reported similar grazing days for cereal rye–annual ryegrass pastures, when either the pastures were nitrogen fertilized or were grown with winter annual legumes. However, in that study, the cereal rye component contributed less to the forage by the mid-winter to the early spring, after which the ryegrass and hairy vetch made the largest contribution until late April, when the hairy vetch became minimal. Fae et al. [8] found that oat–cereal rye mixtures were more optimal for grazing in no-till maize production than annual ryegrass, because the ryegrass did not begin its period of rapid growth until shortly before the maize planting time. Cereal rye was the earliest maturing winter cereal in the studies by Lauriault and Kirksey [22] and Marsalis et al. [29], attaining boot stage in early April, about the same time that grazing began in the present study. The differences in the timing of optimum forage availability of cereal rye and hairy vetch in the present study and those reported by Butler et al. [18] could be due to the competition exerted by annual ryegrass in the Butler study that was not imposed in the present study, as well as environmental influences on maturity. At any rate, the date that the cattle were removed was later in this study than the planting date for sweet maize, which would be of concern for small landholders who may not have land resources for rotation.

### 3.9. Animal Performance

There was no difference between the years or relay intercropping treatments and no interaction for the initial BW of the heifers used in the study. For the relay intercropping treatments, the ADG of the heifers were 0.81 and 0.76 kg d^−1^ for the maize–rye and maize–vetch relay intercropping treatments, respectively (SEM = 0.05, *p* > 0.33). The ADG in the present study was similar to that reported by Butler et al. [18] for cattle grazing annual cool-season grass + N or annual cool-season grass–legume mixtures during April and May (about the same timeframe as the present study) as well as between those treatments for season-long (Nov. through June) grazing. Fae et al. [8] found that actively growing annual cool-season grasses, including the winter cereals, provided sufficient nutrition to achieve satisfactory animal gains, which ranged from 0.76 to 0.86 kg d^−1^ in their study. Otherwise, the differences in ADG across studies conducted during different seasons may be due to an increased maintenance energy requirement for thermoregulation, particularly during cold periods [32].

Newly weaned calves can also gain 0.2 to 0.6 kg d^−1^ while grazing maize residue with CP supplementation [4], which is consistent with the ADG for the autumn grazing [17], but less than ADG reported here for the spring grazing. When intercropped with the sweet maize stover, the actively growing rye and hairy vetch in the present study were sufficient to supply protein (Table 2) and energy for growth by beef yearlings [32] that was greater than the maize supplemented with protein [4]. Additionally, while the rye was grazed to the point of limited availability, based on the post-grazing biomass yield (Table 3), it was not grazed to a point that was detrimental to animal performance, based on ADG [31]. This study demonstrated the feasibility of relay intercropping rye or hairy vetch into sweet maize as a nutritional management scheme for grazing beef calves in the early spring, after the other pastures are depleted and before summer pastures become available.

## 4. Conclusions

The results of this study demonstrate that either cereal rye or hairy vetch can be used to provide higher value forage in the spring when yearling cattle graze sweet maize stover. Intercropping cereal rye or hairy vetch into sweet maize at the maize V7–9 maturity stage did not influence the sweet maize stover biomass yield or nutritive value after winter. However, CP concentration of the hairy vetch biomass was greater than the rye biomass, but the 48-h IVDMD was not different. Average daily gains by yearling cattle were not different when grazing maize–rye or maize–vetch. Producers with limited land resources should consider the timing of the spring planting of the primary crop in relation to the initiation of the grazing of the winter cover crops to maximize the utilization of the previous crop’s residue (stover), as well as the cover crop itself before the time to plant the primary crop. More research is needed to determine if grazing earlier would allow for greater utilization of the maize stover when grazing is deferred until spring, especially if hairy vetch is used as a cover crop to be grazed.

## Figures and Tables

**Table 1:** Monthly mean air temperatures and total precipitation at Alcalde, New Mexico, USA, from each year of the study period and the long-term (1953–2016) means.

Month	Temperature, °C				Precipitation, mm					
	Pre-Year 1 ^1^	Year 1	Year 2	Year 3	Long-Term Mean	Pre-Year 1	Year 1	Year 2	Year 3	Long-Term Mean
January	−1.6	−0.9	0.0	2.3	−0.9	3	12	2	8	10
February	4.5	1.8	1.3	3.9	2.1	15	23	11	1	9
March	5.4	7.4	5.1	7.6	5.8	4	0	22	25	13
April	12.0	8.4	8.7	9.1	10.2	0	25	6	36	15
May	19.3	15.2	15.0	13.3	14.8	0	51	0	22	19
June	21.1	19.2	18.7	18.6	19.6	54	28	7	31	20
July	22.2	21.5	22.8	22.3	22.4	20	37	57	69	35
August	21.4	20.8	21.8	20.2	21.2	42	55	58	89	48
September	15.6	18.4	19.4	16.4	17.0	21	30	16	24	32
October	9.4	10.4	11.0	11.8	10.9	58	10	104	9	26
November	4.2	2.7	5.7	6.2	4.4	4	12	19	0	16
December	−1.5	−1.8	1.9	−1.3	−0.5	10	14	0	4	12
Mean	11.0	10.3	11.0	12.0	10.6	232	298	300	319	254

^1^ Pre-Year 1 represents sweet maize production and interseeding of cereal rye and hairy vetch for grazing in Year 1. Likewise Years 1 and 2 represent the same study set up phases for Years 2 and 3, respectively.

**Table 2:** Relay intercropping treatment main effect means of pre-grazing (mid-April) biomass dry matter (DM) yield, crude protein concentration, and 48-h in vitro dry matter disappearance (48-h IVDMD) of sweet maize stover intercropped with rye or hairy vetch and the overseeded forage species over three years at Alcalde, NM, USA ^1^.

Year	Biomass, Mg DM ha^−1^		Crude Protein, g kg^−1^		48-h IVDMD, g kg^−1 2^	
	Whole Plant Sweet Maize Stover	Over-Seeded Forage	Whole Plant Sweet Maize Stover	Over-Seeded Forage	Whole Plant Sweet Maize Stover	Over-Seeded Forage
1	1.97	1.85	108	176	-----	-----
2	1.78	2.78	97	167	578 ^3,^^a^	497 ^b^
3	1.97	1.97	124	179	531 ^b^	569 ^a^
SED ^4^	0.27	0.50	10	11	14	18
Intercropping Treatment Effect (I)						
Maize–rye	2.02	1.46	110	154	556	548
Maize–vetch	1.79	2.94	110	193	554	518
SED	0.10	0.29	5	6	9	18
***p*** **-Values**						
Year	0.7264	0.2080	0.0940	0.5947	0.0292	0.0041
I	0.0668	0.0024	0.9844	0.0007	0.8926	0.1296
Year × I	0.3970	0.0142	0.9242	0.3386	0.8661	0.0358

^1^ Values in the table are the lsmeans of three replicates for each component of the year × intercropping treatment (Y × I) interaction; ^2^ The statistical analysis and results for 48-h IVDMD only includes two years of data; ^3^ Year lsmeans in the same column followed by the same letter are not significantly different at the 5% alpha level; ^4^ Standard error of the difference between lsmeans.

**Table 3:** Relay intercropping treatment main effect means of post-grazing (late May) biomass dry matter (DM) yield and percentage disappearance of sweet maize stover intercropped with rye or hairy vetch and the intercropping forage species over three years at Alcalde, NM, USA ^1^.

Year	Whole Plant Sweet Maize Stover		Overseeded Forage	
	Biomass, Mg DM ha^−1^	Disappearance, %	Biomass, Mg DM ha^−1^	Disappearance, %
1	0.67 ^2,b^	65 ^a^	0.67	78
2	1.21 ^a^	32 ^b^	0.58	85
3	1.42 ^a^	28 ^b^	0.88	55
SED ^3^	0.20	8	0.46	18
Intercropping Treatment (I)				
Maize–rye	1.18	41	0.41	79
Maize–vetch	1.03	42	1.01	66
SED	0.12	7	0.30	10
	***p*** **-Values**			
Year	0.0274	0.0009	0.8062	0.3037
I	0.2392	0.8150	0.0932	0.2610
Year × I	0.1256	0.3051	0.0795	0.0645

^1^ Values in the table are the lsmeans of three replicates for each component of the year × intercropping treatment (Y × I) effect; ^2^ Year lsmeans in the same column followed by the same letter are not significantly different at the 5% alpha level; ^3^ Standard error of the difference between lsmeans.

## Data Availability

Data are available upon reasonable request from the authors.
